# Evaluating Source Complexity in Blended Milk Cheese: Integrated Strontium Isotope and Multi-Elemental Approach to PDO Graviera Naxos

**DOI:** 10.3390/foods13162540

**Published:** 2024-08-14

**Authors:** Majda Nikezić, Paraskevi Chantzi, Johanna Irrgeher, Tea Zuliani

**Affiliations:** 1 Department of Environmental Sciences, Jožef Stefan Institute, Jamova 39, 1000 Ljubljana, Slovenia; majda.nikezic@ijs.si; 2 Jožef Stefan International Postgraduate School, Jamova 39, 1000 Ljubljana, Slovenia; 3 Interbalkan Environment Center, 18 Loutron Str., 57200 Lagadas, Greece; pchantzi@geo.auth.gr; 4 Union of Agricultural Cooperatives of Naxos, 84300 Cyclades, Greece; 5 Department of General, Analytical and Physical Chemistry, Montanuniversität Leoben, Franz Josef-Straße 18, 8700 Leoben, Austria; johanna.irrgeher@unileoben.ac.at

**Keywords:** ^87^Sr/^86^Sr isotope ratio, ICP-MS, mixing models, dairy production, authenticity tracers, Graviera cheese

## Abstract

Graviera Naxos, a renowned cheese with Protected Designation of Origin status, is crafted from a blend of cow, goat, and sheep milk. This study focused on assessing the Sr isotopic and multi-elemental composition of both the processed cheese and its ingredients, as well as the environmental context of Naxos Island, including samples of milk, water, soil, and feed. The objective was to delineate the geochemical signature of Graviera Naxos cheese and to explore the utility of Sr isotopes as indicators of geographic origin. The ^87^Sr/^86^Sr values for Graviera Naxos samples ranged from 0.70891 to 0.70952, indicating a relatively narrow range. However, the Sr isotopic signature of milk, heavily influenced by the feed, which originates from geologically distinct areas, does not always accurately reflect the local breeding environment. Multi-elemental analysis revealed variations in milk composition based on type and season; yet, no notable differences were found between raw and pasteurized milk. A mixing model evaluating the contributions of milk, sea salt, and rennet to the cheese’s Sr isotopic signature suggested a significant average contribution of 73.1% from sea salt. This study highlights the complexities of linking dairy products with their geographical origins and emphasizes the need for sophisticated geochemical authentication methods.

## 1. Introduction

Historically, concerns about food authenticity have roots in issues such as adulteration and misrepresentation for economic gain [1,2]. As supply chains have become more complex and globalized, the heightened risk of intentional fraud, unintentional contamination, and product mislabeling presents significant challenges with serious implications for public health, consumer trust, and the economic interests of various stakeholders [3]. In addition, ensuring food authenticity is vital for preserving the quality and integrity of food items, as many of them often embody unique characteristics tied to specific regions, reflecting long-standing traditions and distinct production methods [4,5,6]. To safeguard traditional and regionally distinctive foodstuffs, the EU has established quality schemes within the geographical indications system, among which the Protected Designation of Origin (PDO) holds a pre-eminent position [7]. Stringent PDO specifications mandate that every aspect of production, processing, and preparation takes place exclusively in the designated region. In the dairy industry, marked by a broad spectrum of diverse offerings, this designation assumes a crucial role. The pursuit of authentic food represents a collective objective, engaging entities including law enforcement, food producers, traders, and consumers. Importantly, the scientific community holds a pivotal function through the development of advanced analytical techniques [8]. The techniques used for this purpose have primarily centered around light stable isotope fingerprinting in conjunction with trace elemental analysis [9,10,11]. Furthermore, a limited number of studies have explored the application of strontium (Sr) isotope ratios in elucidating the authenticity of these products [12,13,14]. When investigating the chemical signatures in food products, it is crucial to consider a variety of scenarios that might influence the results. Initially, one common scenario involves the direct transfer of the ^87^Sr/^86^Sr isotope ratio from soil to plants and subsequently to the animals feeding on these plants. Notably, during this transfer, the ^87^Sr/^86^Sr ratio typically remains unaltered [15,16,17]. Secondly, human activities, such as irrigation and the use of fertilizers, can significantly alter the Sr isotopic composition of the soil, which subsequently impacts this signature of the plants grown in that soil. Lastly, the isotopic signature of a product can also be affected by various processing and preservation methods, such as salting, which may further alter its original isotopic composition [18]. Understanding these scenarios is essential for accurately tracing the provenance and authenticity of food products, as each factor can significantly influence the interpretative accuracy of isotope-based analyses [19].

By integrating isotope measurements within a geographic framework, complex ecological, geological, and environmental questions can be addressed with unprecedented precision through the use of isoscapes. This approach not only enhances our ability to trace the origins of natural and man-made materials but also bolsters the accuracy of models used to verify the authenticity and provenance of various products in ecological studies. The Aegean region displays a comparatively low variation in Sr isotope ratios. This uniformity could be attributed to a general similarity in the underlying geology of the surveyed regions, primarily composed of limestone and marine sediments. Naxos, however, stands out due to the inclusion of older granites, which are expected to exhibit higher ^87^Sr/^86^Sr values. There are a few significant studies concerning the geographic distribution of Sr isotopes in Greece that use various proxies, and they provide a valuable baseline for all provenance research. The specific details of these studies and their findings are presented in Table 1.

The present study focused on the examination of both milk and cheese samples, specifically the Graviera Naxos PDO variety originating from the island of Naxos, Greece. PDO Graviera Naxos cheese is primarily manufactured in the southwestern region of Naxos, where the prevalent geological formations consist of granodiorite and marble schist. The production of Graviera Naxos cheese occurs at the facilities of the Union of Agricultural Cooperatives of Naxos, utilizing milk sourced from various farms on the island. This hard cheese is crafted from a minimum of 80% cow milk and a maximum of 20% goat and/or sheep milk [26]. The production process adheres strictly to traditional cheese-making practices, incorporating rennet following established guidelines. According to Regulation (EC) No 1107/96 [27] and the Greek national law [28], the milk used for the production of Graviera Naxos must come from cows, goats, and sheep traditionally fed and adapted to the delimited area of production.

This study aimed to link cheese and milk to their environmental sources—feed, soil, and water—through ^87^Sr/^86^Sr isotope ratios and multi-element analysis. Over three years, we tracked seasonal variations in Sr isotopes, established a geochemical database for Graviera Naxos cheese, and assessed the potential of these methods to authenticate dairy product origins.

## 2. Materials and Methods

### 2.1. Reagents and Materials

The preparation of samples and reagents involved the use of ultrapure water (MilliQ) with a resistivity of 18.2 MΩ cm obtained from a Direct-Q 5 purification system (Millipore, Watertown, MA, USA). Nitric acid (67–69% (*w*/*w*) HNO_3_, Suprapur) was sourced from Carlo Erba Reagents Srl (Milan, Italy), while hydrogen peroxide (30% (*w*/*w*) H_2_O_2_, Suprapur), hydrofluoric acid (40% (*w*/*w*) HF, Suprapur), boric acid (H_3_BO_3_, Ultrapur), and ammonium nitrate (≥99.0%, NH_4_NO_3_) were procured from Merck Ltd. (Darmstadt, Germany). Filtration of water and the bioavailable fraction of soil samples was performed using 0.45 μm Minisart cellulose nitrate membrane filters (Sartorius, Goettingen, Germany). The preparation of external calibration for multi-element analysis involved the use of the standard solution Multi VI (ICP Standard Certipur, Merck, Darmstadt, Germany), with accuracy verification conducted using a certified reference material for trace elements in surface water, SPS-SW1 (Spectrapure standards, Oslo, Norway). The following certified reference materials (CRMs) were measured together with the samples during multi-elemental analysis: IAEA 153 (Milk powder, International Atomic Energy Agency, Vienna, Austria), NIST SRM1573a (Tomato leaves, National Institute of Standards and Technology, Maryland, USA), ERM-CC141 (Loam soil (elements), Joint Research Center, Geel, Belgium). For Sr isotope analysis, calibration was performed using NIST SRM 987 standard (strontium carbonate, Gaithersburg, National Institute of Standards and Technology, Maryland, USA). The Supplementary Material (Table S1) provides the average values and standard deviations from the multi-elemental analysis for the appropriate CRMs, as well as the determined limits of detection (LODs) for each element.

### 2.2. Study Area

Naxos is the largest island of the Cyclades (429.79 km^2^), located in the center of the Aegean Sea. The island of Naxos exhibits an elliptical shape, with a primary orientation of N15^ο^E, and consists of metamorphic and igneous rocks of various ages and deformations, allochthonous molasse sediments of the Miocene, and pyroclastic rocks and newer, primarily terrestrial, sediments of the Pliocene-Pleistocene. The greater part of Naxos, especially the eastern and southern section, is occupied by a series of platform metamorphic sediments of Mesozoic age, which were involved in the Alpine orogeny in a lithospheric plate convergence environment. These metamorphic rocks are primarily marbles, interspersed with schists and dolomites, which have a zonal distribution in roughly a NE–SW direction, coinciding with that of the island’s main mountain range (Mount Zeus). The western part of the island is characterized by the appearance of Cycladic I-type granitoids, whose contact with the metamorphic complex is directed N–S, including clays, sandstones, diabases, gabbros, and ultrabasic rocks. Considering the above geographical distribution of geological formations, three main units can be identified on the island: the upper non-metamorphosed unit, the Cycladic Blueschist Unit, and the granodiorite unit [29,30,31,32]. The climate of the study area, although classified as a Mediterranean climate type according to the Thornthwaite climate classification, is dry, and more specifically, xerothermic, with a strong influence of the sea in shaping its thermal character. However, the Aegean Sea, due to its geographical position, numerous islands and peninsulas, bays, and many mountain ranges, as well as other physical and geographical factors, exhibits special meteorological and climatological conditions, to such an extent that a specific type of climate has been established for the Aegean Sea with the term Aegean climate (temperate to maritime) [33]. The annual isotherms range from 18 °C to 19 °C, with a low level of annual rainfall of about 366.80 mm [34]. From a meteorological and climatological perspective, the year is divided into two seasons: the cold and the warm period. The cold period lasts from October to March, while the warm period lasts from April to September. The temperatures prevailing in Naxos in the summer are lower than those in other Greek areas [35]. This is due to the influence of the sea but also the winds with high frequency and intensity, resulting in a reduction in temperature. The prevailing direction of the winds throughout the year is from north to south, and the average annual humidity is about 72%.

### 2.3. Sample Collection and Preparation

**Figure 1:**
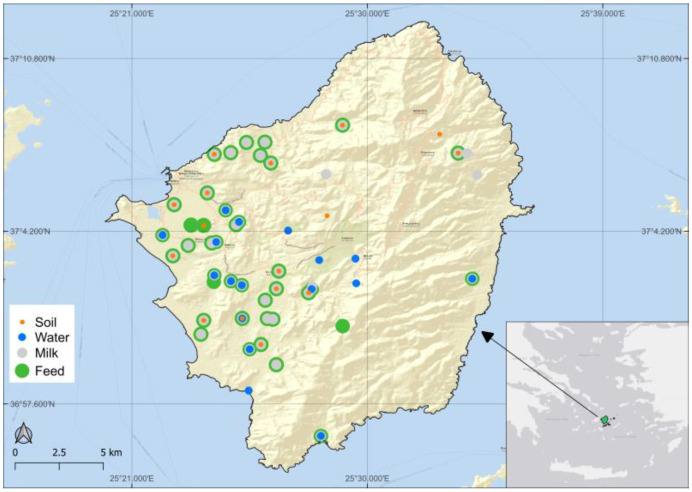
Naxos island and the sampling stations.

Water, soil, feed, milk, and cheese samples were collected from sampling stations on Naxos Island, Greece, as shown in Figure 1. Soil and water were sampled once, in August 2020 and January 2021, respectively. The sampling campaign in August 2020 aimed to study the distribution of Sr isotope ratio values across the entire island, with soil, feed, and milk samples collected from stations #1 to #43. However, our primary focus was on the subsequent campaigns conducted in January 2021 and June 2021, during which both milk and feed samples were collected from identical stations (#51 to #63), allowing for more direct comparisons. Additionally, milk samples were obtained from stations #64 to #66, corresponding to the milk collecting tanks of the cheese-making factory. Furthermore, milk samples were collected in July 2021 and January 2022, all originating from different milk tanks of the cheese-making factory. Comprehensive information and the coordinates of the sampling locations can be found in the Supplementary Material (Tables S2—S7).

*Water samples* (*n* = 18) were obtained mostly from boreholes at depths up to 600 m, as well as from wells and/or springs. Samples originated from stations #51 to #69. After collection, water samples were transferred into clean polyethylene bottles, acidified with suprapure HNO_3_, stored at 4 °C, and then filtered using 0.45 μm nitrocellulose filters before analysis.

*Soil samples* (*n* = 16) were collected at farms using a plastic shovel. They were air-dried and sieved to <63 μm using plastic meshes. To evaluate the content of elements present in the bioavailable form, extraction with 1 mol L^−1^ NH_4_NO_3_ solution was performed following the method described in previous research [24]. Briefly, approximately 2 g of the sample was carefully weighed into a 30 mL test tube, followed by the addition of 5 mL of the extractant. The tubes were then agitated on an orbital shaker for 2 h at 300 rpm. Subsequently, the samples underwent centrifugation at 8000 rpm for 15 min. The supernatant material was separated from the precipitate solution and filtered using a 0.45 μm filter. To guarantee the preservation of sample integrity, each sample was prepared in duplicate and examined on the day of preparation. Additionally, bulk soil samples were subjected to a two-cycle acid digestion in the closed vessel microwave digestion system MARS 6 (CEM corporation, Matthews, NC, USA). Approximately 0.25 g of the sample was weighed into PFA digestion vessels, and a mixture of HNO_3_, HCl, and HF was introduced (4 mL, 1 mL, 2 mL, respectively). The second cycle was conducted following the addition of 12.5 mL of 4% H_3_BO_3_. The resulting clear solution was quantitatively transferred into 30 mL PP graduated conical tubes and filled to the mark with MilliQ water. 

*Feed samples* were garnered in August 2020 (*n* = 36), January 2021 (*n* = 17), and June 2021 (*n* = 14). Each farm used a mixture of different forages, including wheat, bran, corn, barley, soybeans, and cotton seed. In Naxos, the livestock that produce most of the milk for cheese production are typically fed ready-made mixes. This mix primarily consists of 40–50% corn and 15–28% soy. The chemical composition per kilogram of this feed ranges as follows: 14–20.7% total nitrogenous substances, 2.7–3% total fats, 6.50–7.60% fibrous substances, 6–7.4% total ash, 0.8–1.4% calcium, 0.10–0.26% magnesium, 0.25–0.39% sodium, and 0.45–0.70% phosphorus. One feed sample per farm was collected in each sampling campaign. It is noteworthy that while the majority of the samples were these mixtures, a portion of the analyzed samples consisted of independent, single-type-of-grain samples. Some farmers, however, choose to source the raw materials individually and create their own ration mixtures, adhering to the same proportions of corn and soy as in the ready-made mixes. In most instances, these mixtures are prepared approximately, without precise weighing, resulting in a lack of accurate records. Consequently, individual materials were also sampled to verify the range of values. Furthermore, four pasture samples were also gathered for a more comprehensive assessment of forage diversity. Upon arrival at the laboratory, the samples were freeze-dried and subsequently digested in a microwave to prepare them for multi-elemental analysis. For this procedure, 0.30 g of the sample was weighed with the addition of 4 mL of HNO_3_, 1 mL of H_2_O_2_, and 0.1 mL of HF. Afterward, the obtained sample solutions were quantitatively transferred into PP graduated conical tubes and adjusted to a volume of 20 mL with MilliQ water.

*Milk samples* were gathered from various farms over several sampling campaigns: August 2020 (*n* = 32), January 2021 (*n* = 28), June 2021 (*n* = 16), July 2021 (*n* = 10), and January 2022 (*n* = 10). The samples comprise various types, including cow, goat, and sheep milk, as well as blends of these three (tank samples). Moreover, some samples were subjected to pasteurization, while others remained unpasteurized. Milk samples were transported frozen to the laboratory and stored at −80 °C until freeze-drying. For multi-elemental analysis, samples were prepared through a one-cycle microwave digestion. Approximately 0.30 g of freeze-dried milk was introduced into PFA digestion vessels and added with 6 mL HNO_3_ and 1 mL of H_2_O_2_. The resulting solution was quantitatively transferred into PP graduated conical tubes and filled to 20 mL with MilliQ water. 

A total of seven Graviera Naxos *cheese samples* were collected and analyzed. Samples were collected during the following campaigns: August 2020 (*n* = 3), January 2021 (*n* = 1), June 2021 (*n* = 1), and January 2022 (*n* = 2). The two samples received in January 2022 were collected at different times; one sample was fresh uncured, while the other was mature cured cheese. These samples were obtained from the Union of Agricultural Cooperatives of Naxos, where they are produced using milk from distinct farms. For each sampling period, we collected corresponding cheese samples. These cheese samples were taken during the industrial production of milk, rather than under controlled experimental conditions. To ensure that the cheese samples accurately represented the sampling campaign, it was essential to match them with the milk samples. Therefore, we labeled the cheese according to the production date to reflect the characteristics of the milk samples. The cooperative provided us with a single product, as the sampling methodology was destructive to the final product. Cheese samples were processed and prepared using the same methodology as that employed for the milk samples. In addition, sea salt and rennet, used in Graviera Naxos cheese production, were collected and analyzed. Rennet (Naturen Extra 1030 NB, Chr. Hansen Holding A/S, Hoersholm, Denmark) has an instructed dosage of 30–60 IMCU/L of milk, and, as it is in powder form, this means 0.03–0.06 g of powder should be added per liter of milk. Rennet was digested in a similar way as milk and cheese samples. The identical procedure (acids only) was applied to a blank sample in each cycle of digestion of samples of all matrices. In each cycle, the appropriate certified reference material was digested. The measured values for these materials were in alignment with the certified values. A 4% salt solution was prepared by dissolving 2 g of salt in 50 mL of MilliQ water. All samples were diluted 5–10-fold before further analysis. 

### 2.4. Multi-Elemental and Sr Isotopic Analyses

Multi-elemental analysis (Ag, Al, As, B, Ba, Ca, Cd, Co, Cr, Cu, Fe, K, Li, Mg, Mn, Mo, Na, Ni, Pb, Rb, Sb, Se, Sr, Tl, U, V, and Zn) was performed using ICP-MS (7700x series, Agilent Technologies, Tokyo, Japan), with daily optimization of parameters for maximum sensitivity. For Sr isotope ratio analysis, Sr was initially isolated from the matrix through a column procedure employing Sr-specific resin Eichrom® (particle size 100–150 μm, SR-B200-A, TrisKem International, Brittany, France), following the method outlined in previous research [36]. An aliquot of the sample was evaporated to dryness on a sand bath at ≤90 °C, then redissolved in 1 mL of 8 mol L^−1^ HNO_3_, and subjected to ultrasonication for 30 min. Subsequently, the solution was loaded onto a column containing 150 mg of the resin. To assess the efficiency of Rb removal and Sr recovery after separation, the total Sr and Rb concentrations were measured using ICP-MS. Rb concentration was below the limit of detection, and Sr concentration ranged from 15 to 25 ng mL^−1^ in all samples, with a recovery rate exceeding 95%.

The ^87^Sr/^86^Sr isotope ratio was determined using Nu II Plasma MC-ICP-MS (Ametek, Wrexham, UK) equipped with the Aridus II Desolvating Nebulizer System (Teledyne Cetac, Omaha, NE, USA). Instrumental mass discrimination was corrected by internal normalization using the ratio ^86^Sr/^88^Sr = 0.1194, and Rb and Kr interferences were corrected for mathematically using ^87^Rb/^85^Rb and ^86^Kr/^83^Kr ratios of 0.38567 and 1.50566, respectively. All measurements followed the bracketing method (standard-sample–standard sequence) with a sample-matched concentration of the isotope standard reference material NIST SRM 987 (^87^Sr/^86^Sr = 0.71034 ± 0.00026). The instrument underwent daily optimization for maximum sensitivity and signal stability.

### 2.5. Statistical Evaluation

Multi-elemental analysis: Data were expressed as the average ± standard deviation (SD) in the Supplementary Material (Tables S2–S7).

Strontium isotope ratio analysis: The Shapiro–Wilk test showed no significant departure from normality (*p* > 0.05). This finding was further supported by visual inspection of Q–Q plots. However, the Levene test revealed significantly different variances across groups. Given this violation of the homogeneity of variances, the Kruskal–Wallis test was used for comparing means across matrices and within specific matrices, where multiple sampling campaigns occurred. It indicated overall group differences (*p* < 0.05). The Dunn test was applied for post hoc pairwise comparisons. For comparisons involving paired milk and feed samples from the January 2021 and June 2021 campaigns, the Wilcoxon signed-rank test with continuity correction was used. The Dixon test was used for detecting outliers in the datasets. 

Statistical tests and data visualizations were conducted in R Statistical Software [37], using packages such as simmr, ggplot2, ggbeeswarm, among others.

## 3. Results and Discussion

### 3.1. Elemental Composition of Environmental Samples

The content of elements Ag, Al, As, Ba, Cd, Co, Cr, Cu, Fe, Li, Mn, Mo, Ni, Pb, Rb, Sb, Se, Sr, Tl, U, V, and Zn is reported for water, bioavailable soil fraction, and bulk soil samples. Additionally, the same elements, along with Ca, K, Mg, and Na, were measured in feed samples. The Supplementary Material (Tables S2—S7) contains the average values and standard deviations from the multi-elemental analysis across all tested matrices. The analysis included the assessment of potentially toxic elements in each matrix.

For the feed samples, the presence of As, Cd, and Pb was observed in the majority of cases; however, the detected mass fractions remained under the threshold limits established by Directive 2002/32/EC [38]. Expectedly, the contents of these elements were higher in pasture samples than in feed mixture samples. For example, while the average mass fraction of Pb in feed mixture samples was 0.143 mg kg^−1^, in one pasture sample, the Pb mass fraction was measured at 2.14 mg kg^−1^. In terms of water samples, all tested instances showed Cd levels below the LOD, while the measured levels of As and Pb did not surpass the threshold values outlined in the Guidelines for Drinking Water Quality, which are 10 µg L^−1^ and 9 µg L^−1^, respectively [39]. Furthermore, water samples were in accordance with the requirements of the Drinking Water Directive (98/83/EC) regarding As, B, Cd, Cr, Ni, Pb, Sb, and Se concentrations [40]. 

### 3.2. Elemental Composition of Milk and Graviera Naxos Cheese Samples

**Figure 2:**
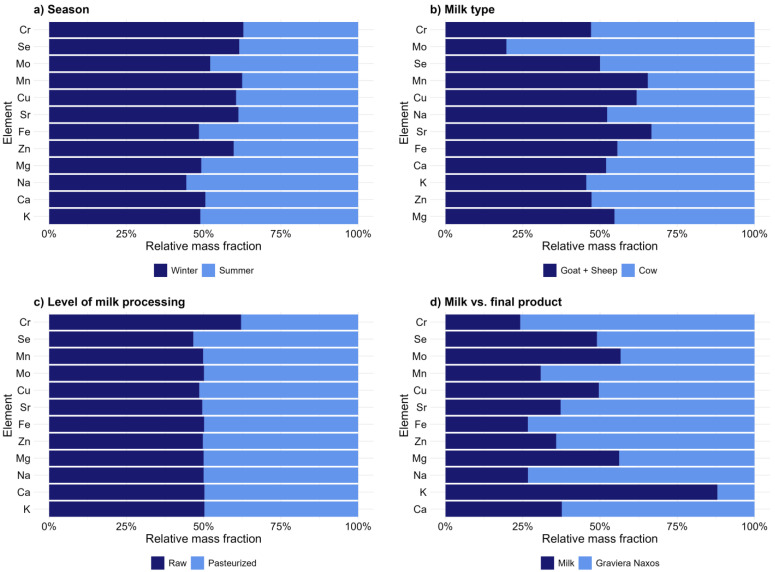
Variations in Sr and essential elements’ levels across (**a**) sampling seasons (January 2021 and June 2021), (**b**) milk types, (**c**) pasteurized and raw milk, and (**d**) milk from milk tanks and Graviera Naxos cheese. The relative mass fraction is normalized to 100% for all elements. Elements are displayed based on their total mass fraction, from lowest to highest.

The content of elements Ag, Al, As, B, Ba, Ca, Cd, Co, Cr, Cu, Fe, K, Li, Mg, Mn, Mo, Na, Ni, Pb, Rb, Sb, Se, Sr, Tl, U, V, and Zn is reported for milk and Graviera Naxos cheese samples. The mass fractions of Ag, Li, Sb, and Tl were below the LOD in all Graviera Naxos cheese samples, while they were detected in approximately 5% of the milk samples. Co, U, and V were above the LOD in approximately 30% of milk and Graviera Naxos cheese samples. On average, macroelements in cow and goat milk were found in the following sequence from lowest to highest mass fractions: Mg < Na < Ca < K, ranging from 739 mg kg^−1^ to 10311 mg kg^−1^, while in Graviera Naxos cheese samples as well as sheep milk samples, mass fractions of Ca were the highest. The content of the microelements in the goat and sheep milk was as follows: Zn > Fe > Cu > Mn > Se > Mo > Cr. However, cow milk samples showed a different pattern, with higher average mass fractions of Mo, rearranging the sequence to Zn > Fe > Cu > Mo > Mn > Se > Cr. In Graviera Naxos cheese samples, the order of microelement content was found to be Zn > Fe > Cu > Mn > Mo > Se > Cr. The comparative analysis of elemental composition indicated that the pasteurization process does not significantly alter the mineral composition of milk, aligning with the conclusions of earlier studies [41]. Additionally, the data revealed less than 25% seasonal fluctuations in the content of most elements (Figure 2a). Comparative assessments further suggest that goat and sheep milk exhibit marginally higher levels of the majority of elements compared to cow milk [42]. Similarly, Graviera Naxos cheese demonstrates a modestly enriched elemental profile compared to its milk precursor (Figure 2d). However, this enrichment does not extend to Mg and K, aligning with previous findings [43]. Given that cheese production involves the mixing of three types of milk, a variance in mineral composition in the cheese is anticipated. Moreover, the creation of dairy matrices during this process leads to unique structures and distributions of essential minerals, distinguishing the cheese from its milk source [44]. In the case of milk, As and Cd mass fractions were found to be below the LOD, and Pb was detected in 17 samples, without exceeding the maximum levels permitted by the European Union, as specified in Commission Regulation (EC) No 2023/915 [45]. The mass fractions of these elements were below the LOD in all Graviera Naxos cheese samples.

### 3.3. Distribution of ^87^Sr/^86^Sr Values and Statistical Analysis

^87^Sr/^86^Sr isotope ratio was determined in soil, water, feed, milk, and cheese samples. Sr isotopic ratio values ranged from 0.70781 to 0.72276 in all analyzed matrices (Table 2). 

^87^Sr/^86^Sr isotope ratio values observed for bulk soil were expectedly high (0.72276), as this is a result of total Sr that is present in all the minerals and organic phases present in the soil. The ranges for the rest of the matrices are characterized by the maximum value of 0.71613 observed in a feed sample. Similarly, it is evident in Table 2 that matrices, except bulk soil, share similar ^87^Sr/^86^Sr isotope ratio means (Water > Bioavailable soil fraction > Feed > Milk > Cheese). 

**Figure 3:**
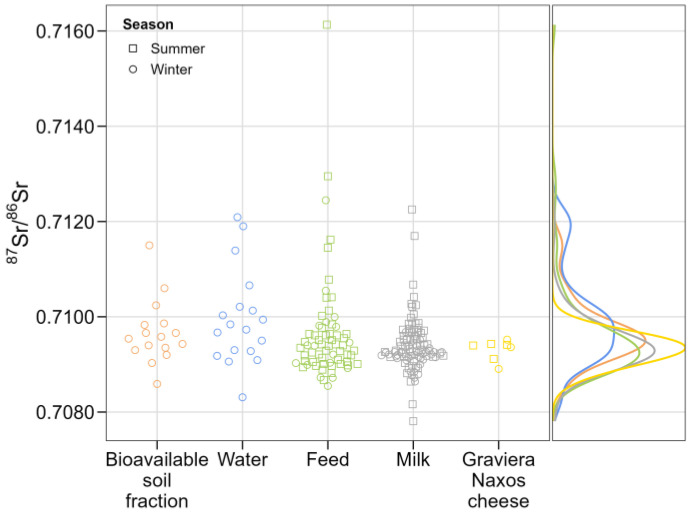
Ranges and distribution of ^87^Sr/^86^Sr values across matrices (KDE bw = 0.0005641063).

Figure 3 displays the ^87^Sr/^86^Sr values across matrices—bioavailable soil fraction, water, feed, milk, and cheese—with each data point representing a distinct sample. This presentation enables the observation of sample density, their respective Sr isotope ratio ranges, and the frequency of sampling during winter and/or summer seasons. Notably, feed and milk samples, collected during summer sampling campaigns, display some of the highest Sr isotope ratio values observed. On the right side of the figure, a Kernel density estimation (KDE) plot is presented, depicting the estimated probability density function of the underlying distribution of ^87^Sr/^86^Sr values across the examined matrices. 

The bandwidth was determined following Scott’s rule of thumb. This data smoothing technique facilitates a closer examination of the local range of bioavailable Sr. Prior to initiating the mixing models, this KDE analysis provides a foundational framework. By visually assessing the positions of the generated peaks, we can infer whether specific sources are likely contributors to the mixture. The primary mode encompasses ^87^Sr/^86^Sr values ranging between 0.7085 and 0.7105, aligning with the bioavailable baseline range for the South Aegean as established by Frank et al. [25]. Nonetheless, the range observed in this study marginally exceeds those previously established boundaries. The cheese matrix peak shows the highest density, indicating the central density of the distribution, as well as the prevalence of these values within the dataset. This is likely due to the smaller number of samples collected and analyzed for cheese compared to feed and milk. Additionally, because cheese is a processed product, its isotopic composition is expected to show less variation compared to milk and environmental samples. The peaks corresponding to cheese, milk, and feed matrices align closely, with each peak falling slightly below the next. Conversely, peaks representing the bioavailable soil fraction and water matrices exhibit a slight upward shift (higher values). Bimodal distribution is observed for the water matrix, suggesting the underlying heterogeneity in the data, probably arising from the presence of multiple distinct sources. Furthermore, no skewness is observed in the distribution, revealing a relatively balanced representation of data around the central mode.

**Figure 4:**
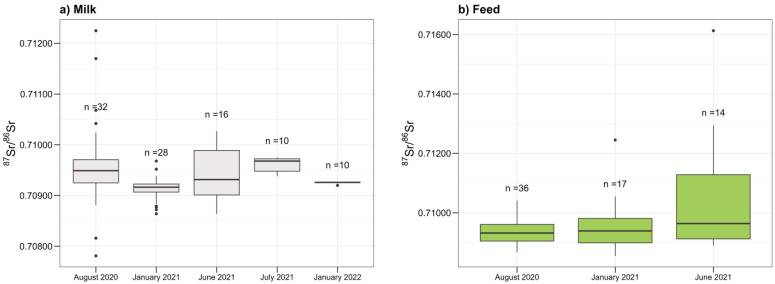
Boxplots representing ranges of ^87^Sr/^86^Sr values for (**a**) milk and (**b**) feed across different sampling campaigns. The median of the data is the line; the points outside the whiskers are points beyond 1.5 times the IQR from the quartiles.

The outcomes of the Dunn test (*p* < 0.05) elucidated notable distinctions in median values among the groups, except between water and bioavailable soil fraction, feed and milk, feed and cheese, and between milk and cheese, where no significant differences were observed. Focusing on the milk matrix, the results, validated by the Dunn test with Bonferroni correction, revealed significant differences in medians between the following campaigns: August 2020 and January 2021, January 2021 and July 2021, as well as July 2021 and January 2022. The Wilcoxon signed-rank test resulted in *p* < 0.05, indicating a significant shift in the medians between January 2021 and June 2021 milk samples. The graphical representation of these findings, as depicted in the boxplots in Figure 4a, best illustrates the observed results. Clearly, the differences were consistently observed between campaigns conducted in the summer and winter seasons. Conversely, comparisons between summer-to-summer or winter-to-winter campaigns did not yield significant differences (*p* > 0.05). Additionally, analogous tests were conducted to compare pasteurized and non-pasteurized milk, as well as various milk types, including cow, goat, and sheep. The results did not show significant differences in the medians of Sr isotope ratio values among these categories (*p* > 0.05). This insight proves to be particularly valuable given that Graviera Naxos cheese is crafted from a blend of various milks. However, the statistical tests performed on feed samples did not provide sufficient evidence to conclude that there is a significant difference in the median values among sampling campaigns (*p* > 0.05) (Figure 4b). 

While it is reasonable to anticipate a significant difference in the medians of Sr isotope ratio values of the samples collected in the summer and winter campaigns, due to seasonal variations in animal diet, namely more pasture grass in summer and limited grazing time in winter, we need to stress that the analyzed feed samples were mostly cereal mixtures—same in both summer and winter. This uniformity in feed composition across seasons could likely explain the inability to detect significant differences in the medians of Sr isotope ratios within the feed samples. In contrast, variations observed in the milk samples, probably influenced by the changing diet of animals, could account for the detection of differences in the medians of Sr isotope ratios between the two seasons. Despite the presence of points outside the whiskers in Figure 4, which likely represent the outliers, the Dixon test identified only one outlier: feed sample with a ^87^Sr/^86^Sr value of 0.71613. However, this value was retained for further analysis, as it was believed to reflect a genuine measurement rather than an error.

### 3.4. Contributions to Sr Isotopic Composition of Milk

Soil, water, and feed samples were investigated as environmental factors likely to influence the Sr isotopic composition of milk. There was no correlation between milk and bioavailable soil fraction samples. Milk samples from August 2020 were used for this comparison, since they originate from the same stations as soil samples. Moreover, the Sr isotope ratio in vegetation primarily reflects the bioavailable Sr fraction of the soil in which it grows [46,47]. Consequently, we investigated the correlation between soil and feed samples. The absence of a correlation between bioavailable soil fraction and feed samples (August 2020) suggests that the feed mixtures were likely not sourced locally from the farms but instead imported to the island. 

**Figure 5:**
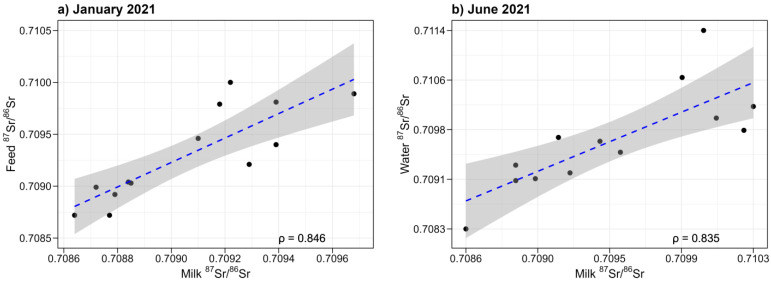
Correlation between ^87^Sr/^86^Sr values of (**a**) milk and feed samples from January 2021 and (**b**) milk (June 2021) and water samples. The line of best fit is represented by a dashed blue line; the shaded area surrounding the line depicts the 95% confidence region.

For the correlation between milk and feed samples, we focused on campaigns conducted in August 2020, January 2021, and June 2021. Despite no significant difference in the medians of ^87^Sr/^86^Sr values of feed samples from August 2020, January 2021, and June 2021, a moderate-to-strong positive correlation emerged between the ^87^Sr/^86^Sr values of milk and feed sampled in January 2021 (Figure 5a). This correlation was quantified by a Spearman correlation coefficient of ρ = 0.85. Interestingly, no obvious correlation was noted for samples from either August 2020 or June 2021. As previously noted, all feed samples, regardless of season, consist of mixtures of various cereals. This suggests that the absence of correlation between milk and feed in August 2020 and June 2021 may be attributed to increased grazing time during this period, leading to a higher proportion of pasture in the animals’ diets. Conversely, during winter, the feed mixtures analyzed constitute a substantial portion of the animals’ diet, resulting in a stronger correlation between milk and feed. 

These statements can be confirmed with further analysis. Namely, the relationship between ^87^Sr/^86^Sr values in milk and water samples was also examined. The values for water and milk from corresponding farms were compared, from both January 2021 and June 2021. No correlation between milk and water ^87^Sr/^86^Sr values from January 2021 was found. However, a moderate-to-strong positive correlation was observed for samples from the June 2021 campaign, as shown in Figure 5b. This is in agreement with another study [48], which also found a correlation between milk and corresponding river samples. The animals’ drinking behavior and water intake are influenced by various factors, including climatic conditions and individual characteristics, such as lactation stage. Despite these influences, the average daily free water intake remains relatively high at 83.6 ± 17.1 liters per day [49]. 

However, water is unlikely to be a major contributor to the Sr isotopic composition of milk due to low Sr concentrations in water samples (0.103 mg L^−1^–2.48 mg L^−1^). Instead, pasture grown on the island, which contains more moisture compared to the dry mixtures provided in winter, may have a similar Sr isotope ratio as water samples from the island. This suggests that during summer, pasture is likely shaping the Sr composition of milk. The Sr isotopic signature of milk is influenced by the diet, significantly altering the ⁸⁷Sr/⁸⁶Sr ratio in milk between seasons, likely masking local Sr isotope ratio levels (water, soil). This finding complicates the use of the ⁸⁷Sr/⁸⁶Sr ratio as a method for verifying the geographical origin of dairy products. In Naxos’s case, where the area for growing feed is limited, and there is a necessity to import feed from the mainland, this is particularly challenging.

### 3.5. Mixing Model for Estimating Contributions to Cheese Sr Isotopic Composition

Mixing model calculations were applied in order to determine the contributions of existing sources to Sr isotopic composition of Graviera Naxos cheese. For this purpose, milk, sea salt, and rennet were defined as possible Sr sources. For fitting the stable isotope mixing model (SIMM) via the Markov chain Monte Carlo (MCMC) algorithm, R package simmr was used [50]. Mixing models have proven critical for detailed investigations. Namely, in the archeological domain, they are indispensable for delineating local bioavailable Sr isotope ranges, facilitating the reconstruction of ancient population migration patterns and geographical origins [51,52,53]. 

In the present model, two tracers were used: ^87^Sr/^86^Sr isotope ratio value and Sr mass fraction. Milk samples obtained from stations 64–66 in January 2021, along with samples collected during the July 2021 and January 2022 campaigns, were used, as these samples were sourced from the milk tanks at the cheese manufacturing facility directly. Additionally, the ^87^Sr/^86^Sr isotope ratio of the analyzed sea salt sample was 0.70928 ± 0.00001. This value aligns with the reported modern seawater ^87^Sr/^86^Sr ratio of 0.7091 [54]. The ^87^Sr/^86^Sr isotope ratio for rennet was determined to be 0.70821 ± 0.00002. To the best of our knowledge, there are no existing reports of ^87^Sr/^86^Sr isotope ratio values specifically for rennet used in cheese production.

**Figure 6:**
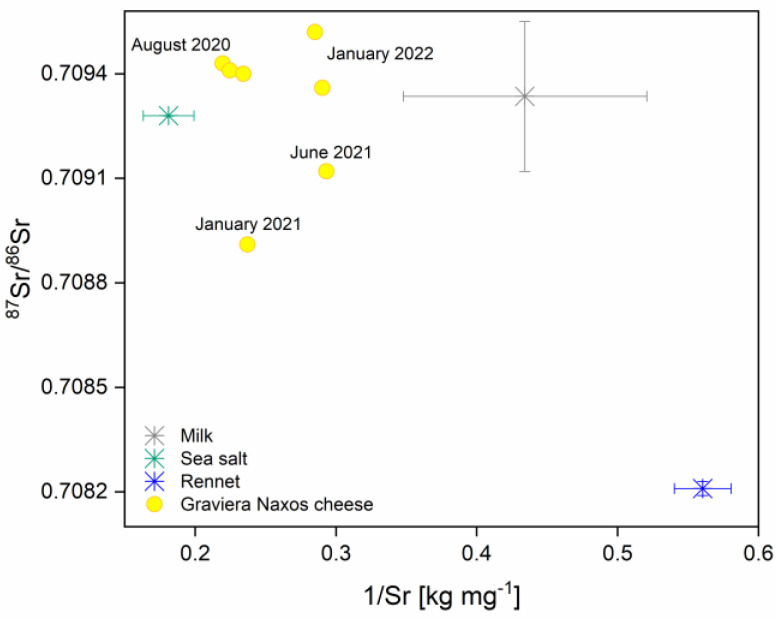
Isospace plot, ⁸⁷Sr/⁸⁶Sr ratios of sources (milk, sea salt, rennet) and Graviera Naxos cheese samples plotted against their respective inverse Sr mass fractions.

The isospace plot (Figure 6) illustrates the positioning of the mixtures relative to potential sources. The observations indicate that the mixtures fall within the polygon formed by connecting the sources with straight lines, suggesting that, likely, no sources were overlooked. After checking the convergence, the model provides the summary that includes the mean and standard deviation estimates for the proportion of each source. Additionally, the three cheese samples from August 2020 are noticeably clustered closely together on the isospace plot. Yet, there is no discernible pattern relating to the season or year of cheese production in their distribution on the plot. Furthermore, it is evident that cheese samples are positioned significantly closer to sea salt and milk sources than to rennet, which is distinctly separate on the plot. Moreover, the larger SD associated with the milk source, in comparison to the other two sources, indicates potential uncertainties in determining source contributions.

All SIMMs use informative prior distributions as a default. The shape and spread of the prior distribution indicate a lower degree of certainty (Figure S1). In the case of sea salt, the posterior distribution differs notably from the prior: the peak has moved closer to the likelihood and become narrower, signifying increased confidence. This means that, on average, sea salt contributes approximately 73.1%, constituting the predominant source influencing the overall Sr isotopic composition of the Graviera Naxos cheese. The estimates for milk and rennet are 15.3% and 11.7%, respectively (Figure S2). 

Figure S3 shows the source histograms, contour plots of the relationship between the sources, and the correlation between the sources. In Figure S3, it can be deduced that there is no observable correlation between rennet and sea salt, and rennet and milk. However, a moderate-to-strong negative correlation exists between sea salt and milk. This suggests that the model struggles to differentiate between the two sources, likely due to their proximity in the isospace (Figure 6) along the y-axis. The provided probability table (Table S8) indicated the probabilities of different mixing scenarios based on the model results. The model strongly supports the hypothesis that sea salt is a major contributor to the Sr isotopic composition of the analyzed cheese samples. The significant influence of salt used in cheese production on its Sr isotopic fingerprint is plausible, especially considering that it typically contains 2.4 g of salt per 100 g of the final product, and the concentration of Sr in seawater lies between 7.2 and 7.8 mg L^−1^ [55]. Some researchers studied the influence of salt on the Sr isotope ratio in cured ham, and their findings indicated that the ^87^Sr/^86^Sr isotope ratio in cured Bayonne ham was strongly influenced by the Sr isotope composition in salt from the local salt mine [56]. However, arriving at a definitive conclusion is challenging due to the close Sr isotope ratio values between sea salt and milk. This suggests that sea spray could significantly affect the entire island’s environment, dispersing onto pastures and consequently impacting the Sr isotope ratio in milk [57]. As demonstrated in Figure 5b, there is a notable correlation between milk and water during the summer, indicating this influence. The mixing model offers a structured approach to understanding the complex mixing scenarios that can occur during cheese production, enabling conclusions to be drawn about the origins of specific isotopic signatures. However, in cases where sources exhibit similar isotopic ratios, such as sea salt and milk in this instance, the model’s effectiveness may be limited. The proximity of isotopic values between these sources poses challenges in accurately distinguishing their contributions, highlighting the need for additional complementary techniques or refined modeling approaches to overcome such complexities. Moreover, the potential influence of salt on the cheese’s Sr isotopic fingerprint suggests the need for further investigation. In addition, having only seven cheese samples limits the statistical significance of the findings, and accuracy could be enhanced with the availability of a larger number of samples for analysis.

## 4. Conclusions

This study represents a significant advancement in the geochemical authentication of dairy products, specifically focusing on the complex matrix of blended milk cheese, such as PDO Graviera Naxos. Through the innovative use of stable isotope mixing models (SIMMs), we were able to dissect the contributions of diverse sources—milk, sea salt, and rennet—to the Sr isotopic composition of cheese. Our findings highlight that sea salt, influenced by local environmental factors, serves as a predominant contributor to the Sr isotopic signature of the cheese, a novel insight that enhances our understanding of geochemical influences in dairy products.

However, the study also highlights several challenges. The close isotopic ratios between different sources, such as sea salt and milk, complicate the distinct delineation of their individual contributions to the cheese’s isotopic profile. This difficulty points to the potential limitations of SIMMs when sources share similar isotopic signatures. Additionally, seasonal variations significantly impact the Sr isotopic composition in milk, suggesting that environmental factors like diet changes and sea spray may mask local isotopic signals, further complicating source attribution.

Future research should aim to refine the application of SIMMs by incorporating additional complementary techniques that can enhance the resolution of isotopic distinctions between sources. Investigating the influence of environmental and processing factors in greater detail will also be crucial. This could involve a more extensive sampling framework that covers a broader range of environmental conditions and processing variables. Ultimately, the goal is to develop a more robust methodological framework that can reliably authenticate the geographical origins of complex dairy products like Graviera Naxos cheese, thereby supporting the protection of PDO products and ensuring product integrity for consumers.

## Figures and Tables

**Table 1:** Chronological summary of past research on ^87^Sr/^86^Sr ratios in Greece, focusing on the South Aegean region and, more specifically, Naxos.

Sample Type	Location	Range of ^87^Sr/^86^Sr Ratio Values	Reference
Whole-rock schist (*n* = 1)	Naxos, Greece (southeast)	0.70930	Andriessen et al. (1979) [20]
Whole-rock granite (*n* = 9)	Naxos, Greece (north)	0.71418–0.72282	
Granodiorite (*n* = 21)	Naxos, Greece (west)	0.71056–0.71724	
Natural mineral water (*n* = 11)	Greece (w/o South Aegean)	0.70782–0.70946	Voerkelius et al. (2010) [21]
Pig and cow enamel, archaeological human bone, modern snail shell (*n* = 8)	Naxos, Greece (central and western island)	0.70903–0.71004	Nafplioti (2011) [22]
Modern hare mandible (*n* = 6)	Naxos, Greece	0.70881–0.71068	Prevedorou et al. (2015) [23]
Grazing and agricultural soil (*n* = 6)	South Aegean, Greece	0.70500–0.71000	Hoogewerff et al. (2019) * [24]
Previous studies (*n* = 28)	South Aegean, Greece	0.70806–0.70972	Frank et al. (2021) ** [25]

* The provided range is an approximation of the ^87^Sr/^86^Sr value ranges for the South Aegean based on spatial modeling. ** The study defined reference baseline for the South Aegean as $\overset{-}{x}$ ± 2σ, using data available from studies [22,23,24].

**Table 2:** Overview of the samples and their ^87^Sr/^86^Sr values.

Matrix	Sampling Campaign	Number of Samples	^87^Sr/^86^Sr				
			Mean	Median	Min.	Max.	SD
Bulk soil	*August 2020*	16	0.71303	0.71182	0.70865	0.72276	0.00373
Bioavailable soil fraction	*August 2020*	16	0.70967	0.70956	0.70859	0.71150	0.00067
Water	*January 2021*	18	0.70996	0.70978	0.70831	0.71209	0.00100
Feed	*August 2020, January 2021, June 2021*	67	0.70963	0.70935	0.70855	0.71613	0.00109
Milk	*August 2020, January 2021, June 2021, July 2021, January 2022*	96	0.70940	0.70926	0.70781	0.71225	0.00058
Graviera Naxos cheese	*August 2020, January 2021, June 2021, January 2022*	7	0.70928	0.70938	0.70891	0.70952	0.00021

## Data Availability

The original contributions presented in the study are included in the article/Supplementary Material, further inquiries can be directed to the corresponding author.

## Supplementary Materials

The following supporting information can be downloaded at: https://www.mdpi.com/article/10.3390/foods13162540/s1, Figure S1: Prior and posterior distributions; Figure S2: Comparison of source proportions; Figure S3: Correlation matrix showing relationships among sources: milk, sea salt, and rennet; Table S1: Average mass fractions and standard deviations obtained through CRMs analyses and LODs for all matrices; Table S2: Description and elemental and Sr isotopic composition of water samples; Table S3: Description and elemental and Sr isotopic composition of bulk soil samples; Table S4: Description and elemental and Sr isotopic composition of bioavailable soil fraction samples; Table S5: Description and elemental and Sr isotopic composition of feed samples; Table S6: Description and elemental and Sr isotopic composition of milk samples; Table S7: Description and elemental and Sr isotopic composition of cheese, rennet, and sea salt samples; Table S8: Most probable orderings of contributions according to the mixing model.
